# Eggshell Membrane Peptides Alleviate IL-1β-Induced Inflammatory Responses and Extracellular Matrix Degradation in Canine Chondrocytes by Inhibiting the NF-κB Signaling Pathway

**DOI:** 10.3390/ani16131939

**Published:** 2026-06-23

**Authors:** Xin Mao, Ling Xu, Yong Cao, Meifeng Wang, Wencan Wang

**Affiliations:** 1 Pet Basic Research and Development Center, Chongqing Sweet Pet Products Co., Ltd., Chongqing 401120, China; 2 Guangdong Provincial Key Laboratory of Nutraceuticals and Functional Foods, College of Food Science, South China Agricultural University, Guangzhou 510642, China; 3 Chongqing Institute for Food and Drug Control, Chongqing 401121, China

**Keywords:** eggshell membrane peptides, IL-1β, canine chondrocytes, inflammatory responses, extracellular matrix, NF-κB signaling pathway

## Abstract

Background: Eggshell membrane peptides (ESMPs) are natural bioactive compounds with reported chondroprotective properties. However, their regulatory effects on canine chondrocytes remain unclear. This study investigated ESMP in an interleukin-1β (IL-1β)-induced inflammatory model of canine chondrocytes. Methods: Chondrocytes were assigned to control (Cont), IL-1β, and ESMP + IL-1β groups. Cell viability was assessed using the Cell Counting Kit-8 (CCK-8) assay. NF-κB p65 nuclear translocation was evaluated by immunofluorescence staining. Real-time quantitative PCR (RT-qPCR) and Western blotting (WB) were used to measure mRNA and protein expression levels, respectively. Results: ESMP inhibited IL-1β-induced NF-κB p65 nuclear translocation and reduced the IL-1β-induced increases in interleukin-6 (IL-6), cyclooxygenase-2 (COX-2), inducible nitric oxide synthase (iNOS), and matrix metalloproteinase-13 (MMP-13) at both mRNA and protein levels. ESMP also decreased IL-6, nitric oxide (NO), and prostaglandin E_2_ (PGE_2_) levels in culture supernatants. ESMP reversed the IL-1β-induced reduction in type II collagen α1 chain (COL2A1) and aggrecan (ACAN) expression at both transcriptional and protein levels. Conclusions: ESMP attenuates IL-1β-induced inflammatory responses and extracellular matrix degradation in canine chondrocytes, potentially associated with suppression of NF-κB p65 nuclear translocation. This supports its potential application in promoting joint health in dogs.

## Simple Summary

Osteoarthritis is a common joint disease in dogs characterized by chronic pain and reduced mobility. Conventional long-term therapies may be associated with adverse effects. This study evaluated eggshell membrane peptides (ESMPs), derived enzymatically from eggshell membranes, in canine chondrocyte inflammation. ESMP treatment was applied following inflammatory stimulation in cultured chondrocytes. The results demonstrated that ESMP effectively alleviated inflammation and supported the maintenance of joint health. ESMP suppressed the activation of NF-κB, a key pro-inflammatory signaling pathway. These results suggest that ESMPs, as a safe and natural daily dietary supplement, may mitigate discomfort and enhance joint health in dogs suffering from osteoarthritis. However, further in vivo studies are required to validate these effects.

## 1. Introduction

Osteoarthritis (OA) is a highly prevalent degenerative joint disease in companion animals, particularly in dogs, which are among the most affected species. Epidemiological studies estimate that approximately 20% of adult dogs and up to 80% of elderly dogs are affected [1]. OA is clinically characterized by chronic joint pain and progressive loss of mobility, resulting in a marked decline in animal welfare and quality of life [2]. Standard therapeutic approaches rely on nonsteroidal anti-inflammatory drugs (NSAIDs) and corticosteroids. However, long-term use of these medications is associated with adverse effects involving the gastrointestinal tract, liver, and kidneys, as well as increased treatment costs [3,4]. Therefore, dietary supplementation with natural bioactive compounds possessing joint-protective properties has gained attention as a safe, sustainable, and cost-effective strategy for OA management.

Eggshell membrane (ESM), a by-product of egg processing, is composed predominantly of protein (~90%) and contains other bioactive components such as keratin, glycosaminoglycans, and lysozyme [5]. Previous research on ESM and its enzymatically hydrolyzed form, eggshell membrane peptides (ESMPs), has primarily focused on clinical and animal studies. Accumulating clinical evidence suggests that ESM and ESMP supplementation can alleviate joint pain and stiffness and improve joint function in OA [6,7,8]. In experimental arthritic rat models, ESM supplementation reduced circulating biomarkers associated with extracellular matrix (ECM) degradation and improved structural joint integrity [9]. Furthermore, ESM has been shown to suppress inflammatory mediators and exert immunomodulatory effects in mice [10]. Veterinary clinical studies further demonstrate that ESM supplementation can reduce joint pain, improve mobility, and enhance quality of life in dogs affected by OA [11,12].

Chondrocytes are the only resident cells in cartilage and are essential for maintaining ECM homeostasis, making them central to OA pathogenesis [13]. Canine chondrocyte models are widely used for in vitro mechanistic studies of OA. Jiang et al. reported that exosomes from canine bone marrow mesenchymal stem cells reduced IL-1β-induced inflammatory responses and promoted anabolic gene expression [14]. Seo et al. demonstrated that polydeoxyribonucleotide suppressed LPS-induced inflammatory cytokine secretion via modulation of the NF-κB pathway [15]. Similarly, Kim et al. showed that platelet-rich plasma modulated ECM-related gene expression and reduced TNF-α levels in LPS-stimulated canine chondrocytes [16]. These studies confirm the suitability of IL-1β-stimulated canine chondrocytes as a reliable model for screening potential chondroprotective agents.

Research on ESM/ESMP in chondrocyte biology remains limited. Previous studies have shown that ESM protects human chondrocytes from H_2_O_2_-induced oxidative stress [17]. Moreover, ESMP exhibits well-documented antioxidant and cytoprotective properties [18,19,20], and in vitro studies indicate that ESMP reduces excessive reactive oxygen species levels and mitigates ECM degradation in human chondrocytes [21]. In canine models, the ESM protein has been reported to reduce oxidative injury and maintain chondrocyte phenotype [22]. However, the molecular mechanisms underlying ESMP-mediated regulation of IL-1β-induced NF-κB activation and subsequent ECM imbalance in canine chondrocytes remain unclear. Bioactive peptides derived from animal sources, including bovine, seahorse, and fish, have been reported to exert significant chondroprotective effects such as anti-inflammatory activity, pain reduction, and inhibition of ECM degradation [23,24,25,26,27,28,29,30]. Based on these findings, ESMP is hypothesized to exert protective effects in canine chondrocytes by inhibiting NF-κB p65 nuclear translocation and restoring ECM balance.

Therefore, this study was designed to evaluate ESMP in an IL-1β-stimulated canine chondrocyte model, focusing on NF-κB signaling and ECM regulation. NF-κB p65 nuclear localization and inflammatory and ECM-related markers were assessed to elucidate the mechanistic role of ESMP. These findings may support the potential application of ESMP as a functional ingredient for canine joint health.

## 2. Materials and Methods

### 2.1. Materials and Reagents

ESMP was supplied by Milai Biotechnology Co., Ltd. (Wuhan, China). Dulbecco’s Modified Eagle Medium/Nutrient Mixture F-12 (DMEM/F-12), fetal bovine serum (FBS), and 0.25% trypsin-EDTA were obtained from Haixing Biosciences (Suzhou, China). PBS, penicillin/streptomycin solution, BSA, RIPA lysis buffer, and TBST were purchased from Coolaber Science & Technology (Beijing, China). Paraformaldehyde (4%), 0.1% Triton X-100, PBST, DAPI, and antifade mounting medium were obtained from Biosharp (Beijing, China). Immunofluorescence blocking solution was purchased from Beyotime (Shanghai, China), and canine IL-1β protein was acquired from MedChemExpress (Shanghai, China). Anti-NF-κB p65 (WL01980) and anti-ACAN (DF7561) primary antibodies were obtained from Wanleibio (Shenyang, China) and Affinity Biosciences (Nanjing, China), respectively. Primary antibodies against GAPDH (60004-1-Ig), COX-2 (27308-1-AP), iNOS (22226-1-AP), MMP-13 (83188-4-RR), and COL2A1 (28459-1-AP), together with CoraLite^®^ Plus 488-conjugated goat anti-rabbit secondary antibody (RGAR002), were obtained from the Proteintech group (Wuhan, China).

### 2.2. Molecular Weight (MW) Distribution Analysis of ESMP

ESMP was prepared as a 2 mg/mL filtered solution and separated by high-performance liquid chromatography (HPLC) on a TSKgel G2000SWXL column (7.8 mm × 300 mm, Tosoh Bioscience, Tokyo, Japan) at 25 °C with a 10 μL injection volume. The mobile phase consisted of acetonitrile, water, and trifluoroacetic acid (20:80:0.1, *v*/*v*/*v*) delivered with a flow rate of 0.5 mL/min and UV detection at 220 nm. MW calibration was performed using five reference standards: cytochrome c (12,327 Da), aprotinin (6512 Da), bacitracin (1422 Da), Gly-Gly-Tyr-Arg (451.5 Da), and Gly-Gly-Gly (189.2 Da). A calibration curve (Y = −0.2292x + 6.809, R^2^ = 0.9971) was constructed by regressing log-transformed MW values against retention times of the standards. The MW ranges were calculated from the ESMP retention times, and the relative percentages of each MW fraction were determined from peak areas.

### 2.3. Cell Culture

A canine chondrocyte line (Global Pets’ Cell Resource Center, Tianjin, China) at passage 2 was maintained in T-25 flasks in DMEM/F-12 medium supplemented with 10% FBS and 1% penicillin/streptomycin solution at 37 °C under 5% CO_2_. All subsequent experiments were initiated upon cells reaching 80% confluence.

### 2.4. Cell Viability Assessment

Cell viability was assessed using the Cell Counting Kit-8 (CCK-8, Boxbio Science & Technology Co., Ltd., Beijing, China). Cells were seeded at 5 × 10^4^/mL (100 μL/well) in 96-well plates and cultured for 24 h. The medium was removed, and the cells were subsequently exposed to ESMP at concentrations of 0, 0.5, 1, 2, 5, 10, and 20 mg/mL for another 24 h. CCK-8 reagent (10 μL/well) was then added, plates were incubated for another 2 h, and absorbance was measured at 450 nm using a microplate reader (Diatek, Wuxi, China).

Furthermore, cells in the ESMP + IL-1β group were pre-treated with 2 mg/mL ESMP for 2 h following the initial 24 h culture period and then stimulated with 10 ng/mL IL-1β for 24 h. No pre-treatment was administered to cells in the Cont and IL-1β groups. CCK-8 detection followed the same procedure described above.

### 2.5. Immunofluorescence Staining

Cells grown in 12-well plates were pre-incubated with or without 2 mg/mL ESMP for 2 h and then stimulated with or without 10 ng/mL IL-1β for 30 min. After three PBS washes, cells were fixed in paraformaldehyde for 20 min, permeabilized with 0.1% Triton X-100 for 6 min, and incubated with blocking solution for 1 h at room temperature. Cells were then incubated with anti-NF-κB p65 primary antibody (1:300) for 16 h at 4 °C, washed three times with PBST, and incubated with CoraLite Plus 488-conjugated goat anti-rabbit secondary antibody (1:500) for 1 h at room temperature in the dark. Following three additional PBST washes, nuclei were stained with DAPI (1 μg/mL) for 5 min, and cells were mounted with antifade mounting medium. Three fields of view per sample were examined under a fluorescence microscope (Yongxin Optics Co., Ltd., Ningbo, China). The nucleus-to-cytoplasmic ratio of the fluorescence signal was quantified using ImageJ software (v1.54g) to achieve semi-quantitative analysis of p65 nuclear translocation.

### 2.6. Biochemical Assays and Enzyme-Linked Immunosorbent Assay (ELISA) for Supernatant Mediator Quantification

Following designated treatments, cell culture supernatants were collected to quantify nitric oxide (Beijing Boxbio Science & Technology Co., Ltd., Beijing, China) and prostaglandin E2 (Solarbio Science & Technology Co., Ltd., Beijing, China) concentrations using commercial detection kits. IL-6 concentration was determined by ELISA (Shanghai Kexing Trading Co., Ltd., Shanghai, China) according to the manufacturer’s instructions.

### 2.7. Real-Time Quantitative PCR (RT-qPCR)

Total RNA was extracted and purified using the RNApure Total RNA Kit (Aidlab Biotechnologies Co., Ltd., Beijing, China). Complementary DNA (cDNA) was synthesized using the M-MLV 4 First-Strand cDNA Synthesis Kit with gDNA Eraser (Beijing Biomed Gene Technology Co., Ltd., Beijing, China), and RT-qPCR was performed using qPCR MasterMix (Beijing Biomed Gene Technology Co., Ltd., Beijing, China). Amplification conditions consisted of an initial denaturation at 95 °C for 2 min, followed by 40 cycles of 95 °C for 20 s, 60 °C for 15 s, and 72 °C for 30 s. Primer sequences are listed in Table 1 and were synthesized by Tsingke Biotechnology Co., Ltd. (Beijing, China). *GAPDH* served as the internal reference gene, and relative mRNA expression levels were calculated using the 2^−∆∆CT^ method [31].

### 2.8. Western Blotting (WB)

Cells in 6-well plates were washed three times with PBS and lysed in RIPA buffer supplemented with 1% PMSF on ice for 30 min. The resulting lysates were centrifuged at 12,000 rpm for 15 min at 4 °C, and total protein concentration was determined using a BCA assay kit (Cobio Biotechnology Co., Ltd., Wuhan, China). Protein samples (50 μg/lane) were resolved by SDS-PAGE on 8%, 10%, or 12% gels at 120 V and transferred onto PVDF membranes at 300 mA. Membranes were blocked with 3% BSA in TBST for 30 min and then incubated with primary antibodies against COX-2, iNOS, MMP-13, COL2A1, and ACAN (all at 1:1000) for 16 h at 4 °C. Following five washes with TBST, membranes were incubated with secondary antibody (1:5000) for 30 min at room temperature. Protein bands were visualized using an ECL chemiluminescence kit (Beyotime, Shanghai, China) and imaged on a ChemiScope 6100 system (Clinx Science Instruments, Shanghai, China). Band intensities were quantified using AlphaEaseFC software (v4.0), normalized to GAPDH and expressed as fold change relative to the control group.

### 2.9. Statistical Analysis

All experiments were performed in three independent replicates. Data are presented as mean ± SEM. Group differences were assessed by one-way ANOVA with post hoc testing using SPSS software (v21.0). The LSD test was applied when variances were homogeneous; Dunnett’s T3 test was used when variance homogeneity was violated. Figures were generated using GraphPad Prism (v10.6.1). Statistical significance was defined as *p* < 0.05.

## 3. Results

### 3.1. Molecular Weight (MW) Distribution of ESMP

The MW distribution of ESMP is presented in Table 2. HPLC analysis indicated that peptides with MW below 3000 Daltons (Da) accounted for more than 77% of total ESMP content. Peptides below 500 Da were the most abundant fraction at 47%, followed by those in the 500 to 1000 Da and 1000 to 3000 Da ranges at 16.53% and 14.2%, respectively.

### 3.2. Effects of ESMP on Cell Viability

**Figure 1:**
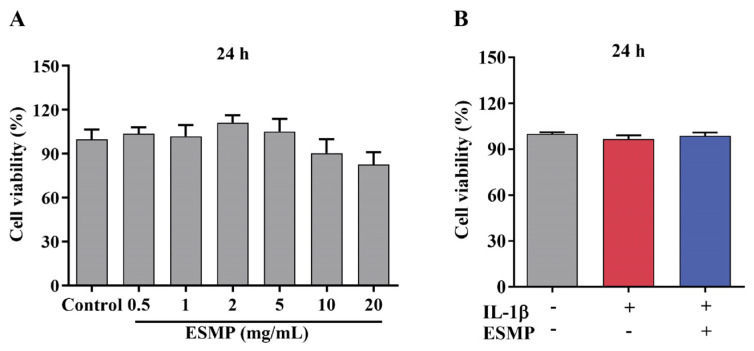
Cell viability assessment using the CCK-8 assay. (**A**) Viability of chondrocytes following exposure to varying concentrations of ESMP. (**B**) Viability of chondrocytes after 24 h treatment with IL-1β alone or in combination with ESMP. Data are presented as mean ± SEM (*n* = 3).

CCK-8 assay results showed that ESMP at concentrations of 0.5–20 mg/mL exerted no significant effect on canine chondrocyte viability (Figure 1A, *p* > 0.05). Cell viability peaked at 2 mg/mL and declined at higher concentrations (Figure 1A). Therefore, 2 mg/mL of ESMP was used for subsequent experiments. Neither IL-1β alone nor in combination with ESMP significantly altered cell viability relative to the Cont group following 24 h treatment (Figure 1B, *p* > 0.05).

### 3.3. ESMP Inhibits IL-1β-Induced Nuclear Translocation of NF-κB p65

**Figure 2:**
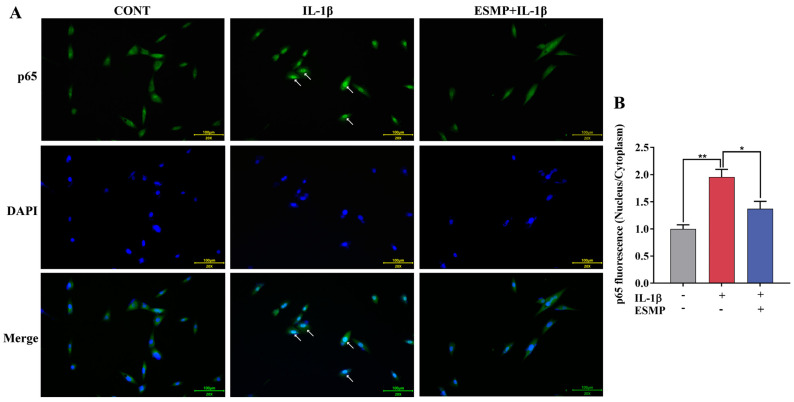
Immunofluorescence analysis of NF-κB p65 localization. (**A**) Representative immunofluorescence images showing the subcellular distribution of NF-κB p65 (green) in canine chondrocytes. Nuclei were counterstained with DAPI (blue). The white arrows indicate nuclear translocation of p65. Magnification: 20×; scale bar = 100 μm. (**B**) Quantitative analysis of the nucleus-to-cytoplasm ratio of p65 fluorescence intensity. Data are presented as mean ± SEM (*n* = 3). * *p* < 0.05 and ** *p* < 0.01.

In the Cont group, p65 fluorescence signals were predominantly localized in the cytoplasm. IL-1β stimulation induced a marked increase in nuclear accumulation of p65, accompanied by enhanced nuclear fluorescence intensity. ESMP treatment significantly reduced nuclear p65 accumulation, indicating inhibition of p65 nuclear translocation (Figure 2A). Quantitative analysis of fluorescence intensity showed that the nucleus-to-cytoplasmic ratio of p65 was significantly increased following IL-1β stimulation and was subsequently decreased after ESMP treatment (Figure 2B, *p* < 0.01).

### 3.4. ESMP Suppresses IL-1β-Induced Upregulation of Inflammatory Mediators

**Figure 3:**
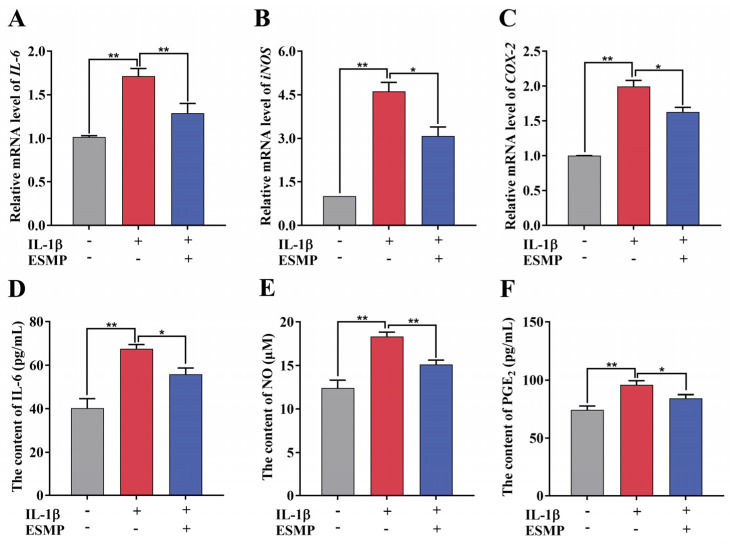
ESMP suppresses IL-1β-induced upregulation of inflammatory mediators in canine chondrocytes. (**A**–**C**) mRNA expression levels of *IL-6*, *iNOS*, and *COX-2*. (**D**–**F**) Levels of IL-6, NO, and PGE_2_ in the cell culture supernatant. Data are presented as mean ± SEM (*n* = 3). * *p* < 0.05 and ** *p* < 0.01.

Exposure of chondrocytes to IL-1β significantly increased the mRNA expression levels of *IL-6*, *iNOS*, and *COX-2* (Figure 3A–C, *p* < 0.01), as well as the levels of IL-6, NO, and PGE_2_ in the culture supernatant (Figure 3D–F, *p* < 0.05).

**Figure 4:**
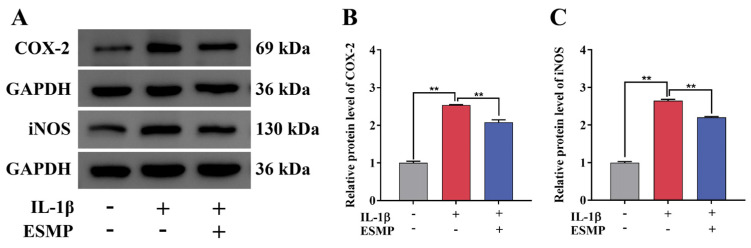
ESMP suppresses IL-1β-induced upregulation of inflammatory proteins in canine chondrocytes. (**A**) Representative Western blot images showing target proteins and GAPDH as a loading control. (**B**,**C**) Relative protein expression levels of COX-2 and iNOS. Data are presented as mean ± SEM (*n* = 3). ** *p* < 0.01.

WB analysis showed a significant increase in COX-2 and iNOS protein expression following IL-1β treatment (Figure 4A–C, *p* < 0.01). ESMP treatment significantly reversed the IL-1β-induced upregulation of these inflammatory proteins (Figure 4, *p* < 0.01).

### 3.5. ESMP Restores IL-1β-Induced Imbalance in ECM Metabolism in Chondrocytes

**Figure 5:**
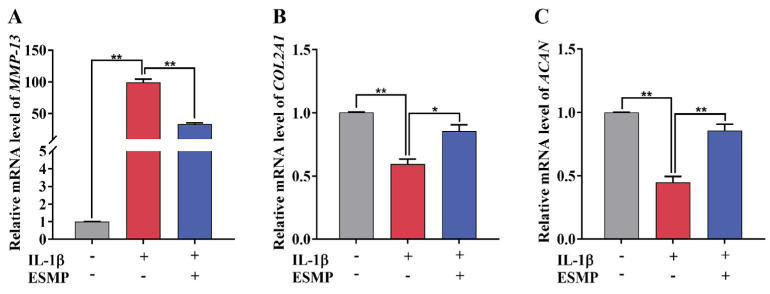
Effects of ESMP on IL-1β-induced expression of ECM-related genes in canine chondrocytes. (**A**–**C**) mRNA levels of *MMP-13*, *COL2A1*, and *ACAN*. Data are presented as mean ± SEM (*n* = 3). * *p* < 0.05 and ** *p* < 0.01.

To further evaluate the effects of ESMP on ECM homeostasis in chondrocytes, ECM metabolism-related markers were analyzed. IL-1β stimulation significantly increased *MMP-13* mRNA expression by nearly 100-fold compared with the Cont group, which was subsequently reduced following ESMP treatment (Figure 5A, *p* < 0.01). IL-1β significantly decreased the mRNA expression levels of *COL2A1* and *ACAN,* whereas ESMP treatment restored their expression (Figure 5B,C, *p* < 0.01).

**Figure 6:**
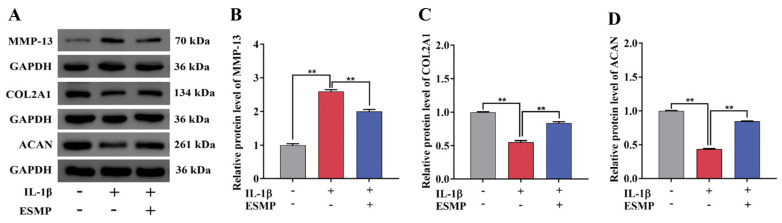
Effects of ESMP on IL-1β-induced expression of ECM-related proteins in canine chondrocytes. (**A**) Representative Western blot images of target proteins and GAPDH as a loading control. (**B**–**D**) Relative protein expression levels of MMP-13, COL2A1, and ACAN. Data are presented as mean ± SEM (*n* = 3). ** *p* < 0.01.

Consistent with the mRNA expression data, similar trends were observed at the protein level for MMP-13, COL2A1, and ACAN (Figure 6A–D, *p* < 0.01). ESMP treatment reversed the IL-1β-induced downregulation of COL2A1 and ACAN and attenuated the upregulation of MMP-13 at the protein level (Figure 6, *p* < 0.05).

## 4. Discussion

This study systematically evaluated the chondroprotective effects of ESMP against IL-1β-induced inflammatory injury in canine chondrocytes. The findings demonstrated that ESMP effectively inhibited IL-1β-induced nuclear translocation of NF-κB p65, accompanied by significant downregulation of pro-inflammatory mediators (IL-6, iNOS, and COX-2) and the catabolic enzyme MMP-13. ESMP significantly reversed the IL-1β-induced suppression of COL2A1 and ACAN expression, restoring disrupted ECM homeostasis.

Animal-derived bioactive peptides exhibit a wide range of physiological regulatory activities, including antibacterial [32], antioxidant [33], cardiovascular protective [34], immunomodulatory [35], and OA-alleviating effects [36,37,38,39]. Low-MW peptides (<3 kDa) are widely recognized for superior cellular bioavailability and enhanced biological activity [40,41]. Consistent with previous reports showing that collagen-derived peptides below 3 kDa enhance chondrocyte anabolic activity and suppress MMP-13 overexpression in multiple chondrocyte models [42,43], the ESMP used in this study was predominantly composed of peptides below 3 kDa. This compositional profile further supports its potential to attenuate inflammation and regulate ECM metabolism in canine chondrocytes. Subsequent cytotoxicity evaluation using gradient concentrations confirmed the favorable biocompatibility of ESMP within the 0–20 mg/mL range, with no evident cytotoxic effects. Although differences in cell viability among treatment groups were relatively small, the dose-dependent curve presented a characteristic bell-shaped pattern, with maximal viability observed at 2 mg/mL. This response profile is commonly reported for natural bioactive compounds in chondrocyte-related studies [44,45,46]. Considering optimal cellular vitality and to minimize non-specific effects associated with high concentrations, 2 mg/mL was selected as the working concentration for all subsequent experiments.

The NF-κB signaling pathway is a central regulatory axis in the pathogenesis and progression of OA [47,48]. Upon IL-1β stimulation, IκB is phosphorylated and subsequently degraded, leading to the release of the p65 subunit, which translocates into the nucleus to initiate transcription of downstream pro-inflammatory genes [49,50]. Ishikawa et al. reported that canine p65 shares more than 87.5% sequence homology with its mammalian counterparts, confirming its evolutionary conservation and supporting the suitability of canine chondrocytes as a reliable model for NF-κB-related OA research [51]. Excessive nuclear accumulation of p65 can establish a self-amplifying catabolic feedback loop, where ECM degradation products, such as hyaluronan fragments, further enhance p65 nuclear translocation and MMP-13 transcription, accelerating cartilage degradation [52]. In agreement with canine-based mechanistic studies, plant-derived extracts have been shown to suppress IL-1β-induced NF-κB activation by inhibiting IκBα phosphorylation, IκBα degradation, and p65 nuclear translocation [53]. These findings support the concept that targeting p65 nuclear translocation represents a viable strategy to interrupt inflammatory signaling and ECM catabolic progression. Consistent with previous in vitro studies [14,54], the present results confirmed robust activation of NF-κB signaling following IL-1β stimulation, whereas ESMP treatment significantly suppressed p65 nuclear translocation and reduced *IL-6, iNOS,* and *COX-2* mRNA expression. These results suggest that the anti-inflammatory effects of ESMP are partly mediated through inhibition of p65 nuclear translocation, consistent with mechanisms reported for various bioactive compounds in chondrocytes [55,56,57,58,59].

Articular cartilage integrity is maintained by the ECM, which is primarily composed of type II collagen and proteoglycans [60,61]. MMP-13 is a key catabolic enzyme responsible for ECM degradation [62,63,64], and its overexpression is consistently observed in IL-1β-stimulated chondrocytes, alongside suppression of COL2A1 and ACAN expression [44,46,65,66,67]. In canine chondrocytes, Jiang et al. reported a marked increase in *MMP-13* mRNA accompanied by reduced *COL2A1* and *ACAN* transcription following IL-1β stimulation, although corresponding protein-level changes were not fully characterized [14]. In this study, *MMP-13* mRNA expression increased by nearly 100-fold after IL-1β exposure, reflecting strong transcriptional activation driven by inflammatory signaling. This pronounced induction can be attributed to multiple NF-κB response elements within the *MMP-13* promoter, which enable rapid and amplified transcriptional responses to pro-inflammatory stimuli [68,69,70]. Furthermore, IL-1β-induced NF-κB activation enhances c-Fos and c-Jun expression, which cooperatively bind to the *MMP-13* promoter and further potentiate its transcriptional activity [71,72,73]. NF-κB-dependent cytokine signaling amplifies *MMP* expression through positive feedback mechanisms, exacerbating ECM degradation [74,75]. Previous studies have demonstrated that ESMP protects human chondrocytes from oxidative stress by downregulating MMP-13 and restoring COL2A1 protein expression [21]. The present findings extend these observations by confirming that ESMP reverses IL-1β-induced MMP-13 overexpression and restores COL2A1 and ACAN protein levels in canine chondrocytes, indicating a conserved matrix-protective effect across species.

IL-1β-induced inflammatory stimulation significantly upregulates iNOS and COX-2 expression in chondrocytes from mice, rats, and dogs, leading to excessive production of NO and PGE_2_ [65,66,76]. As key inflammatory mediators, NO and PGE_2_ further promote MMP-13 expression and accelerate ECM degradation [77,78,79,80]. It has been reported that NO donors suppress *SOX9* mRNA expression in rabbit chondrocytes [81]. SOX9 is a master transcription factor regulating COL2A1 and ACAN expression [82], and its downregulation impairs ECM synthesis. Furthermore, NO inhibits prolyl hydroxylase activity in chondrocytes [83], an enzyme essential for hydroxylation and stabilization of collagen triple-helix structures, further disrupting collagen biosynthesis. ECM degradation products can reactivate NF-κB signaling, establishing a destructive feedback loop that enhances NO and PGE_2_ production and perpetuates cartilage degeneration [84]. Consistent with these mechanisms, the results demonstrate that ESMP significantly reduces IL-1β-induced NO and PGE_2_ accumulation, which likely contributes to restoration of ECM metabolic homeostasis.

Although this study was performed using an in vitro canine chondrocyte model, the identified chondroprotective mechanisms of ESMP may have translational relevance for other veterinary species, particularly horses. Athletic and aged horses exhibit a high incidence of OA due to chronic mechanical overload [85], while long-term NSAID and corticosteroid use is associated with adverse effects such as gastric ulceration and laminitis [86], underscoring the need for safe nutritional interventions. Given the high evolutionary conservation of NF-κB p65 across mammalian species and similar IL-1β-induced inflammatory responses in equine chondrocytes characterized by elevated MMP-13, iNOS, and COX-2 [87,88,89], the inhibitory strategy targeting p65 nuclear translocation observed in canine chondrocytes may also apply to equine OA management. However, in vivo validation in equine models is required to confirm cross-species applicability.

Despite these strengths, several limitations should be acknowledged. First, this study was conducted in a conventional two-dimensional (2D) monolayer culture system, which may induce chondrocyte dedifferentiation and does not fully recapitulate the native cartilage microenvironment. Three-dimensional (3D) culture systems better preserve chondrocyte phenotype and ECM synthesis capacity [90]. Future studies utilizing 3D hydrogel cultures or cartilage explant models are warranted to validate these findings under more physiologically relevant conditions. Second, the precise molecular target of ESMP within the NF-κB signaling cascade remains unclear. Since NF-κB activity is regulated at multiple levels, including receptor activation, IKK complex signaling, and IκBα phosphorylation [91], future studies employing IKK inhibitors, phosphorylation assays, and siRNA-mediated gene silencing are required to identify the direct molecular target of ESMP. Third, although the MW distribution of ESMP was characterized, the specific peptide sequences and bioactive components remain unidentified. As peptide bioactivity is highly dependent on amino acid sequence and structural motifs [92,93], further peptidomic analyses are necessary to identify active fractions and establish structure–activity relationships.

Another important limitation is the gap between in vitro findings and in vivo efficacy. Although ESMP demonstrated clear chondroprotective effects in vitro, its therapeutic potential has not been validated in animal models of canine OA. Oral administration is the most practical route for veterinary nutritional foods; however, the gastrointestinal stability of ESMP remains uncharacterized. Low-MW peptides (<3 kDa) and proline-rich sequences often exhibit resistance to gastric digestion, and small peptides can be efficiently absorbed via PepT1 transporters in the intestine [94,95]. Moreover, clinical studies in humans have demonstrated that orally administered hydrolyzed ESM improves OA symptoms [8,96], supporting its bioavailability and translational potential. Future studies will focus on validating ESMP in 3D chondrocyte systems and cartilage explant models, followed by systematic in vivo studies in canine OA models to evaluate oral efficacy and therapeutic potential.

## 5. Conclusions

**Figure 7:**
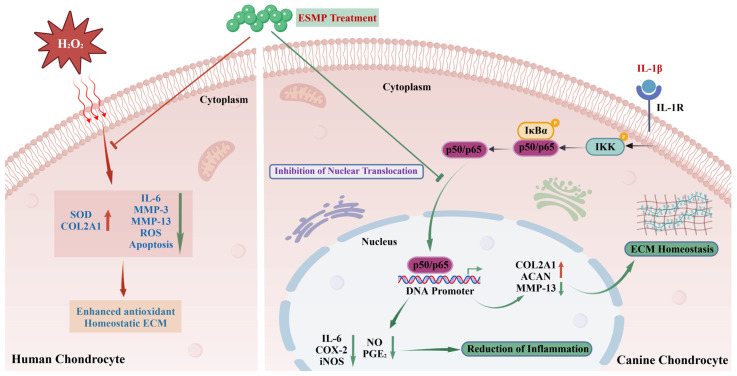
Schematic illustration of the proposed protective mechanism of ESMP in chondrocytes. The diagram summarizes findings from this study (**Right panel**) alongside previously reported effects in human chondrocytes (**Left panel**). The illustration was created using BioGDP.com [97].

This study demonstrates that ESMP exhibits significant in vitro anti-inflammatory and chondroprotective effects in IL-1β-stimulated canine chondrocytes. ESMP suppresses NF-κB signaling by inhibiting the nuclear translocation of p65. This modulation is associated with reduced expression of catabolic mediators, including MMP-13, NO, and PGE_2,_ alongside the preservation of anabolic markers such as COL2A1 and ACAN (Figure 7), contributing to the restoration of ECM homeostasis in canine chondrocytes. As a sustainable byproduct of the egg processing industry, ESMP represents a potentially cost-effective and naturally derived candidate for supporting joint health in dogs, particularly in aging populations, and may hold value as a functional nutritional component in veterinary applications.

## Figures and Tables

**Table 1:** Primer sequences for RT-qPCR.

Gene Name	Sequences (5′→3′)	Tm (°C)	Size (bp)	Accession ID
*GAPDH*	F: GTCCCCACCCCCAATGTATC	60	128	NM_001003142.2
	R: GTGTAGCCCAGGATGCCTTT			
*IL-6*	F: TCCTGGTGATGGCTACTGCTT	60	78	NM_001003301.1
	R: GACTATTTGAAGTGGCATCATCCTT			
*iNOS*	F: AATGGAGAGTTGGGCCTTCC	60	227	NM_001313848.2
	R: TGGCCCTTAAGAGAAGACTGG			
*COX-2*	F: TGTGTCTCATTAACCTGCATGTACC	60	115	NM_001003354.1
	R: CAGTGATATTTGCACCTGTGTCCTC			
*MMP-13*	F: CCTCCCGCGACCTTATCTTC	60	155	XM_038536351.1
	R: CCCCGTGTCCTCAAAGTGAA			
*COL2A1*	F: CAGCGAGCGTTCCCAAGA	60	158	NM_001006951.1
	R: CAGGCGGAGGAAGGTCAT			
*ACAN*	F: ATCAACAGTGCTTACCAAGACA	60	122	NM_001113455.3
	R: ATAACCTCACAGCGATAGATCC			

**Table 2:** Molecular weight distribution of ESMP.

MW (Daltons, Da)	Content (%)
>10,000	11.12 ± 0.54
5000–10,000	6.09 ± 0.21
3000–5000	5.07 ± 0.14
1000–3000	14.20 ± 0.09
500–1000	16.53 ± 0.03
<500	47.00 ± 0.55

## Data Availability

The data presented in this study are available in the manuscript.
